# Cases in the Practice of an Hospital Physician

**Published:** 1814-08

**Authors:** 


*For the Medical and Physical Journal.*

### Cases *in the* Practice *of an* Hospital Physician.

*(Continued from p. 32.)*

### *Case of Eruptive Affection.*

M. C. aged 45, admitted the 14th of January. About three weeks ago a pustular eruption of a particular kind appeared in every part of her body, though not in any considerable quantity. Previous to its appearance, she had been complaining more or less for five or six weeks of various febrile symptoms, such as head-ach, thirst, heat, vomiting, or looseness, and during all these times, every morning, she fell into a profuse sweat, which continued several hours, though without being preceded by any sense of coldness or trembling. All these complaints, she says, were greatly relieved when the eruption appeared, which is now on the decline in most parts of her body, but still continues on her face. Before she was affected with any of these complaints, she had got a fall, by which her shoulder and leg of one side were much hurt, and still continue very painful and numbed; has at different times laboured under the fluor albus. Her menses have not appeared for several years; belly regular; P. 84.

15th.—P. 88. Capt. hor. som. Solut. Merc. Corros. ℥ fs. & Bibt. Decoct. Burdan ℔ij. in die.

19th.—Pustules continue on her face, but go off on the rest of her body; belly loose. Omit. Solut. Sumat. Tinct. Opii, gtt. xxx. o. n.

24th.—Still continues to purge, otherwise complains only of her limbs. Contin. Haust. Anodyn. & inung. humerus & brach. Lin. Ammon. Let her have a flannel sleeve to cover her arm and shoulder.

26th.—Pustules not quite gone off her face; has had two stools to-day, and in other respects as yesterday. Contin. Anod.

29th.—Complains of pains of her arm and leg as before; otherwise well, and without purging; has some œdematous swelling of both her legs. Rep. Haust. Anod. Solut. Tart. Emet. ℥j.

Feb. 1st.—Was sick after the draught last night, but did not vomit; did not sleep so well as formerly, seemingly from the pain of her limbs; sweated some, and has since had a stool. Rep. Haust. Tinct. Opii, gtt. l.

3d.—P. 80, full; complained more of her pains last night, and does so still; had little sleep, and did not sweat last night. Rep. Haust.

6th.—Pains somewhat easier, though her left shoulder is now in some degree affected, and the eruption continues on most parts of her body as fresh as at first. Rep. Haust.

7th.—Pains continue in the right side as before; belly regular; was hardly at all affected with her draught last night; neither sleeps nor sweats much. Rep. Haust. Tinct. Opii, gtt. lx.

9th.—The pains of her limbs were severe last night, and she has had no sleep or sweat. Rep. Haust.

10th.—The pain of her ancle being severe, a blister was applied to it last night, which has risen well, and relieved the pained part; rested better in the night; all her pains easier. Rep. Haust.

11th.—Does not feel the pain of her leg, and has little in her shoulders; slept and sweated some last night. Contin. Haust.

13th.—Has still so much pain of her ancle that she cannot stand upon it. Interior. Malleol. Appr. Emp. Episp. Contin. Haust.

17th.—Pains easier, but much troubled with a cough. Vesp. Capt. Pulv. Ipecac. ℈j. & Hor. Som. Capt. Haust. Anod. ex Tinct. Opii, gtt. xl.

20th.—Cough easier; slept better last night. Contin. Haust.

21st.—Upon getting up she feels the pain of her limbs; slept little in the night, but sweated freely; cough continues; belly regular. Contin. Haust.

23d.—P. last night 106, just now 84; sweated a good deal last night; cough almost gone; pains easier. Contin. Haust.

24th.—Complains of her pains, but more of her stomach, and has had a frequent vomiting of her food for some days past; belly regular. Capt. Pulv. Ocul. Cancror. ℈i. bis in die. Contr. Haust.

25th.—P. frequent, and a little hard; last night was seized with coldness and shivering, and this morning an erysipelas appears upon her face, with much swelling, and already some blisters; belly regular. Mitt. Sang. ad ℥ viij. Let her face be well dusted with flour. P. M. Inj. Enem. Salin. ex Aq. tepid. ℔ifs. Capt. Mist. Nitros. ℥j. 2da q. q. hora.

26th.—P. 104. Erysipelas continues; blood sizy; complains much of sickness; pulse still very full; had a stool by the clyster. Mitt. Sang. ad ℥ viij. Contin. Mist. Nitros. Rep. Enem.

27th.—P. 110, still full; swelling rather fuller than yesterday, though not spreading, and in several places oozes out a moisture; breathes and swallows quite easily; has had a looseness for two days, and therefore did not get the clyster yesterday; yesterday; blood very sizy. Rep. Venæsect. ut heri. Contin. Mist.

28th.—P. 116; slept none last night, and was a little delirious; has had several stools. Vesp. Capt. Solut. Tart. Emet. ℥ ſs. ad 4tam vicem. Con. Mist. Nitros.

29th.—P. 112, not so full or hard as before; erysipelas abating on one side of her face, but continues on the other; slept a little in the night, and was less delirious; vomited after the solution; belly still loose; and takes no food. Rep. Solut. Contin. Mist. Nitros.

March 1st.—P. 100; swelling of her face abating; slept well last night, and feels herself easier, and has taken a little food to-day. The solution did not make her vomit, but has given her several stools. Omit. Sol. et Cont. Mist.

2d.—P. 110, soft and regular; the swelling in general still continues to abate, except on one cheek, where it is rather increased; sleeps almost constantly, and none of her complaints are worse, though she still appears to be confused at times; she has taken more food to-day than for several days past, and with some appetite; diarrhœa, in a moderate degree, still continues. Contin. Mist.

3d.—P. 76; continues to recover; the inflammation on her cheek diminishing; diarrhœa abating. Contin. Mist.

4th.—P. 78; the inflammation of her face quite gone; sleeps a great deal, but is sensible when awake; takes food; belly regular. Contin. Mist.

20th.—Without complaint. Dismissed cured.

### *Case of Hysteria.*

A. M. aged 20, admitted the 10th of October, complains of head-ach, difficulty of breathing, frequent dry cough, pain of her left side which is greatly increased by the cough or making a full inspiration, nausea, loss of appetite, great thirst, and a pain in the small of her back; tongue clean and moist; skin warm; belly natural. Her menses are regular, but they stopped at their last return sooner than usual: this, however, was fourteen days ago, and her complaints came on only last Friday. She can assign no cause for them; has been blooded, and used some medicines with a little relief. Sum. Solut. Antim. Tart. ℥ ſs. om. ½ hor. ad 6tam vicem vesp.

11th.—P. 88. Solution did not make her vomit, but procured three stools. She has frequent startings, occasioned, as she says, by the pain of her side, which is sore to the touch, and she cannot lie on it. When she was first taken ill she was seized with a sort of a fit, as it appeared, quite insensible; says she is unable to speak during this attack, but but hears what is said by the by-standers. After fetching two or three deep sighs, she recovers; says she has had one of these fits every night since Friday; sleeps very ill. Sum. P. Ipec. gr. xv. & sum. Tinct. Opii, gt. xxv. h. s.

12th.—P. 102; vomit operated well; tongue moist and clean; a remarkable exacerbation of her complaints comes on about five or six o'clock in the evening; was restless in the night.

℞. Pulv. Cort. ℈j.

Syr. Simp. q. s. fiat Bol. hor. 4ta vespertin. sumend.

Let her legs be fomented for half an hour together between four and five o'clock in the afternoon.

13th.—P. 76; had no fainting fit last night, and finds herself easier to-day. Rep. Bol. & Fot. ut antea.

14th.—P. 72; had a slight fainting fit last night, but slept well, and has no particular complaint to-day. Cont.

15th.—P. 72; had no fainting fit last night, but complains much of a pain in her left side to-day.

℞. Magn. Alb. ʒj.
Pulv. Zinzib. gr. x. M. Sumat hor. som.
App. Emplast. Galbani part. dolent. later.

16th.—P. 80; had no fainting fit last night; great pain of her stomach, flatulency, and oppression of breathing; appetite a little better. Int. Bol.

17th.—P. 88 by the fire; has had no fainting fit; finds relief from the medicine, which makes her pass a great deal of wind; all her complaints easier. Cont. Med.

18th.—P. 86; complains of head-ach; catamenia have come on since yesterday.

19th.—P. 86; catamenia stopt last night; complains much of the pain at her stomach. Sumat Mist. ex Tinct. Cast.

20th.—P. 80. Had two fits yesterday evening, though she took the castor mixture twice. She was ordered, if threatened with another, to take ℥j. of the mixture with Tinct. Opii, gtt. xv. which, however, did not prevent the accession of a fit about eleven o'clock last night. Complains much of a pain of her head and stomach. App. Emp. Lyttæ Nuch.

21st.—P. 92; had several fainting fits yesterday afternoon; head-ach better; breathes more freely. Cont.

22d.—P. 78; had several fainting fits last night, though she took the castor mixture as usual; complains of great head-ach and pain of her stomach.

℞. Aq. Menth. Pip. ℥j.

Magn. Alb. ʒiſs.

Pulv. Zinzib. gr. x. M. Sum. Statim.

23d.—P. 64; complained of violent head-ach and great oppression of breathing last night, which were relieved by pediluvium, and she fell asleep; complains still of great head-ach, thirst, sickness, loss of appetite, and pain of her stomach. Sum. Pulv. Ipecac. gr. x. Vesp. Tinct. Opii, gtt. xxv. h. s.

24th.—P. 60, vomit operated well, but she threw up only some viscid phlegm; slept well, and complains only of a pain in her stomach. Utatur Vesp. semicup. Calor. 90 Far.

25th.—P. 70; had a pretty good night, and is better to-day.

27th.—P. 90 by the fire; head-ach better; no complaint, but a slight pain in her side. Let the side be rubbed with camphor liniment, and covered with flannel.

30th.—Dismissed cured. Let her have some of the Linim. Ammon. and the other medicines along with her.

### *Case of Jaundice.*

S. J. aged 30, admitted the 2d of March, about three weeks ago was seized with febrile symptoms, which till this time seemed to have recurred irregularly, leaving her altogether for some days. About four days ago, after a more remarkable attack, (which was attended with more severe head-ach, greater sickness, pain and swelling of her stomach, inclination to vomit, drowsiness, and sluggishness) she observed a yellowness of her skin, which has since been increasing, though it is not considerable. The albuginea of her eyes is considerably turgid; for three years past she has been subject to a pain of her right side, which was supposed to have been occasioned originally by carrying a heavy load; this she says is now very severe. By her account she has likewise of late had frequent attacks of erysipelas on her body. Her present illness is imputed to vexation; is commonly costive, but had a stool yesterday, which, she says, was white. Her urine tinges linen yellow. Is a married woman, but never bore children. Her menses have been frequently obstructed, and they have not appeared for seven weeks past. P. 78.

3d. P. 60. Vesp: Capt: Pulv: Ipecac: gr. xv.

4th. P. 72; vomit operated well last night; symptoms continue as before; costive. Inj: Enem: Salin:

℞. Pulv: Summit: Absynth: ℥ ſs.

Rhæi: Elect: ʒj.

Sapon: Hisp: ℥j.

Syr: Com: q. s. fiat Elect: cujus

Capt: Magnitud: Nuc: Mosch: bis in die.

8th. Symptoms as before. Contin: Elect: & vesp: rep: Ipecac: ut antea.

9th. The vomit did not operate freely, and she has had no no stool since; complains of head-ach alternating with pains of her stomach; jaundice symptoms as before. Contin: Elect: Cras mane Capt: Chryst: Tart: ℥i. Pulv: Jallap, gr. xv.

11th. Continues to complain of head-ach, with some chilliness, but her pulse is natural; less jaundice. Vesp: Capt: Pulv: Ipecac. ut antea cras mane. Contin: Elect:

12th. P. natural; vomit operated well last night; jaundice continues to go off; still complains of her head and stomach; has had no stool since the night before last. Contin: Elect: hod: & Capt: Cryst: Tart: ʒj. Omn: ½ hor: ad 3am vicem.

15th. Jaundice quite gone; the Cr: Tart: has not operated yet; complaints of her head and stomach continue. Cr: man: rep: Cryst: Tart. ad Dos: vi.

16th. P. 92; had a severe head-ach and a febrile accession yesterday afternoon, but did not sweat; head-ach and pain of her side still frequently troublesome; the Cr: Tart: has not yet operated.

19th. Has still head-ach, and frequent fits of chilliness, especially in the afternoon; has had no stool these two days.

℞. Fol: Sen: ʒj.

Semin: Corriand: ʒj.

Affund: Aq: Bullient: ℥viij.

Digere per hor: iv. & Colatur: add: Sal: Glaub: ʒvi.

Syr: Limon: ℥j.

Capt: cras: man.

22d. Without complaint. Dismissed cured.

(To be continued.)

